# Safety, tolerability, and pharmacokinetics of HRS9432(A) injection in healthy Chinese subjects: a phase-I randomized, double-blind, dose escalation, placebo-controlled study

**DOI:** 10.1128/aac.00524-24

**Published:** 2024-06-20

**Authors:** Xin Yan, Yuanyuan Huang, Jinlian Xie, Qian Wu, Shuang Yang, Xiaoyan Yang, Honghui Chen, Jie Huang, Guoping Yang

**Affiliations:** 1 Xiangya School of Pharmaceutical Sciences, Central South University, Changsha, Hunan, China; 2 Jiangsu Hengrui Pharmaceuticals Co, Ltd, Shanghai, China; 3 Clinical Pharmacology Center, The Third Xiangya Hospital of Central South University, Changsha, Hunan, China; University of Iowa, Iowa City, Iowa, USA

**Keywords:** HRS9432(A), echinocandin-class antifungal drugs, pharmacokinetics, safety, tolerability, dose escalation

## Abstract

HRS9432(A) is a long-acting echinocandin antifungal medication primarily used to treat invasive fungal infections, particularly invasive candidiasis. The safety, tolerability, and pharmacokinetic characteristics of HRS9432(A) injection were investigated in a randomized, double-blind, placebo-controlled, single- and multiple-ascending-dose Phase I study involving 56 healthy adult subjects. Doses ranging from 200 to 1200 mg were administered. Safety was continually monitored, including adverse events, clinical laboratory examinations, vital signs, 12-lead electrocardiograms, and physical examinations, while the pharmacokinetic profile within the body was evaluated. The results indicated that concentrations of HRS9432 peaked immediately after infusion, demonstrating essentially linear pharmacokinetic characteristics within the dosage range of 200–1,200 mg. It exhibited a low clearance rate and an extended half-life, with a clearance of approximately 0.2 L/h, a volume of distribution of around 40 L, and a half-life of approximately 140h following a single dose. The accumulation index for AUC0-τ after multiple doses ranged from 1.41 to 1.75. No severe adverse events occurred during the study, and the severity of all adverse events was mild or moderate. Therefore, the intravenous administration of HRS9432(A) in healthy Chinese adult subjects, either as multiple infusions of 200 to 600 mg (once a week, four doses) or as a single infusion of 900–1,200 mg, demonstrated overall good safety and tolerability. The pharmacokinetic exhibited essentially linear characteristics in the body, supporting a weekly dosing frequency for clinical applications and providing additional options for the treatment or prevention of invasive fungal infections.

**CLINICAL TRIALS:**

This study is registered with the International Clinical Trials Registry Platform as ChiCTR2300073525.

## INTRODUCTION

Among opportunistic or conditionally pathogenic fungi, invasive candidiasis (IC) emerges as the most prevalent invasive fungal infection, characterized by its high incidence, elevated mortality rate (up to 50%), and substantial diagnostic and therapeutic costs (1). As the population of immunocompromised individuals continues to rise, the demographic at high risk for invasive fungal infections has expanded (2). Therefore, the use of effective antifungal treatments and preventive measures is of paramount importance.

In clinical practice, commonly used systemic antifungal medications include azoles, polyenes, and echinocandins. Azoles and polyenes are associated with severe adverse effects related to hepatic and renal toxicity, and a substantial number of fungal strains have developed resistance to these agents, necessitating the quest for alternative therapeutics to combat fungal infections (3). Echinocandins represent a novel class of antifungal drugs characterized by potent antimicrobial activity, absence of cross-resistance with other drugs, and minimal adverse effects. They disrupted the fungal cell wall structure by non-competitive inhibition of β-1,3-d-glucan synthase, leading to an imbalance in intracellular osmotic pressure, ultimately causing fungal cell lysis and death. Additionally, echinocandins inhibit filamentation in most Candida species and demonstrate fungicidal activity against the majority of Candida species and fungistatic effects against most Aspergillus species. Both the Infectious Diseases Society of America (IDSA) and the European Society of Clinical Microbiology and Infectious Diseases (ESCMID) recommend echinocandin-class antifungal drugs as first-line treatment for invasive candidiasis, second-line treatment for invasive aspergillosis, and the preferred empiric treatment in critically ill patients suspected of having candidiasis in the intensive care unit (ICU).

Echinocandin-class antifungal drugs currently available on the market include caspofungin, micafungin, and anidulafungin. The chemical structures of these drugs contribute to their relatively short half-life, necessitating daily administration in clinical practice (3). Rezafungin is a long-acting echinocandin antifungal drug, based on the structure of anidulafungin to enhance metabolic stability. The elimination half-life after a single intravenous dose is extended to 150 h, significantly longer than other echinocandins on the market, enabling once-weekly dosing (4). The results of its Phase III clinical trial (ReSTORE) indicate that once-weekly dosing of 400 mg/200 mg of rezafungin is comparable to caspofungin in terms of efficacy and clinically manageable in terms of safety. Currently, rezafungin has received FDA approval for marketing (5).

Injectable HRS9432(A) is a long-acting echinocandin antifungal drug jointly developed by Jiangsu Hengrui Pharmaceuticals Co., Ltd. and Fujian Sendi Pharmaceuticals Co., Ltd. Sharing a chemical structure similarity with Rezafungin, this medication prolongs the elimination half-life *in vivo*, significantly reducing the administration frequency. However, the large molecular weight of echinocandins and their poor oral bioavailability have resulted in a lack of oral formulations, increasing attention on long-acting intravenous echinocandin formulations, especially for outpatient or discharged patients. This situation makes daily intravenous injections inconvenient and long-term treatment difficult. Therefore, the development of HRS9432 provides broader therapeutic options for clinical treatment or prevention of invasive fungal infections, while also enhancing treatment convenience and patient compliance. This study represents the first human investigation of injectable HRS9432(A), meticulously monitoring the safety and tolerability of a single intravenous infusion in healthy subjects, while also evaluating its pharmacokinetic characteristics in humans.

## RESULTS

### Characteristics of the subjects

**Fig 1:**
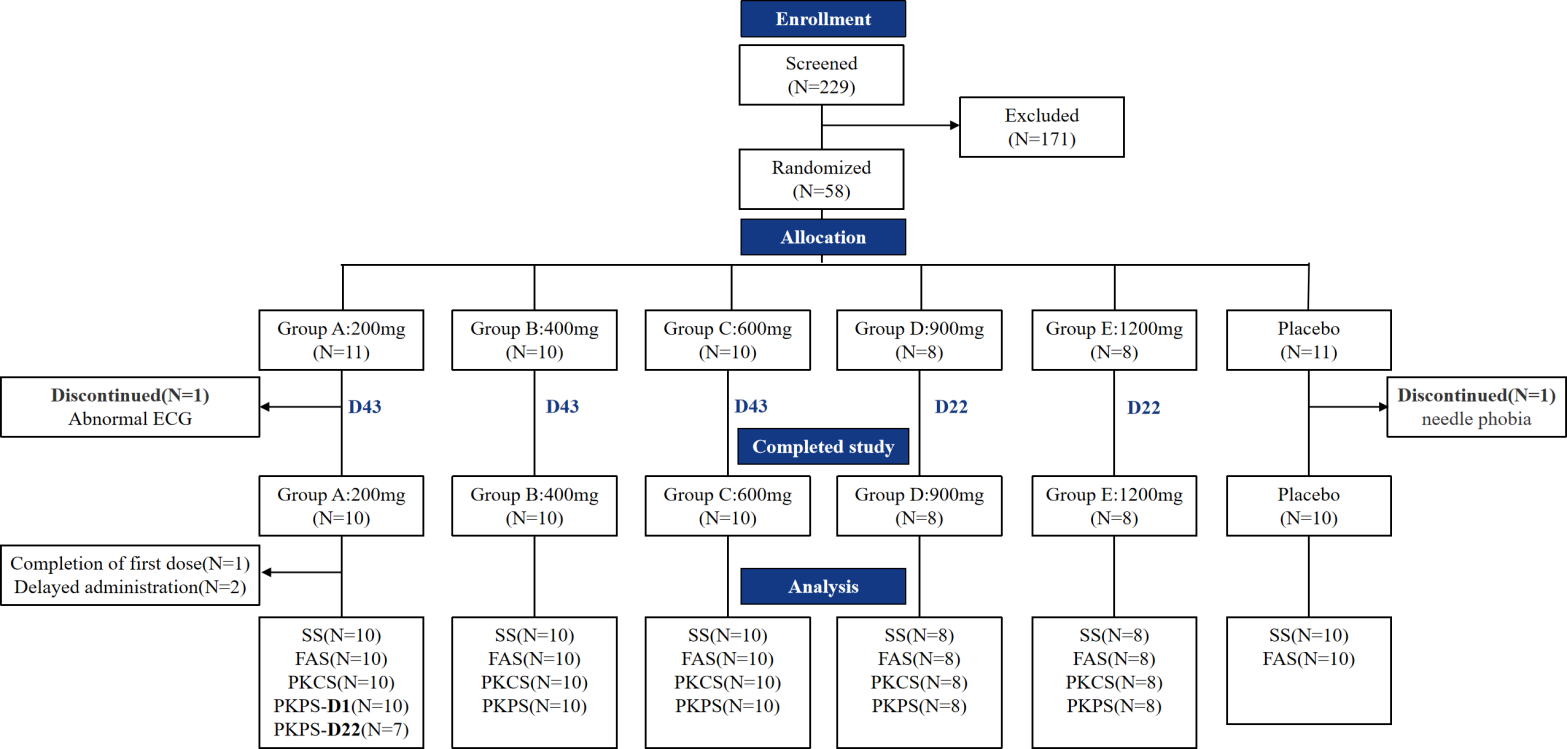
Disposition process of subjects. Safety set (SS), all the subjects enrolled in the study were included in the safety set; pharmacokinetic concentration set (PKCS), all subjects with at least one drug concentration result; pharmacokinetic parameter set (PKPS), all subjects that had at least one viable PK parameter; ECG, electrocardiogram; D1, first day of the study; D22, day 22 of the study.

In this study, a total of 58 subjects were enrolled, with two subjects discontinuing the trial before receiving medication. Of the 56 subjects included in the full analysis set (FAS) and safety analysis set (SS), 46 subjects from the treatment group, with the exception of the 10-placebo group subjects, were included in the pharmacokinetic concentration set (PKCS) and PKPS. During the course of the study, one subject withdrew from the trial, resulting in a total of 55 subjects who received medication and completed the trial. Two subjects had a 7-day delay in their third dose administration due to a third occurrence of COVID-19 infection, which had a significant impact on their PK evaluation. Consequently, the concentration and PK data from before and after the third and fourth doses for these two subjects were excluded from the overall descriptive summary analysis, PK linear assessment, steady-state analysis, and gender-based differences analysis. Figure 1 shows disposition process of subjects.

Of the 56 subjects included in the FAS, 28 were males and 28 were females, making up 50.0% each, with 46 of them being of Han ethnicity. The average age was 27.1 ± 6.06 years, with a mean height of 164.02 ± 8.91 cm, an average weight of 60.71 ± 8.98 kg, and a mean BMI of 22.50 ± 2.20 kg/m^2^. Demographic baseline information was well-balanced among the groups. Additional details are provided in Table 1.

**TABLE 1:** Demographics and baseline characteristics (full analysis set)^*a*^

	A: 200 mg (*N* = 10）	B: 400 mg (*N* = 10）	C: 600 mg (*N* = 10）	D: 900 mg (*N* = 8）	E:1200 mg (*N* = 8）
Age (years）					
Mean (SD)	26.1 (5.67)	27.4 (6.62)	30.2 (6.56)	24.3 (4.23)	24.9 (4.19)
Median	24.5	24.5	29.5	24.0	24.0
Sex, *n*(%）					
Male	5 (50.0)	5 (50.0)	5 (50.0)	4 (50.0)	4 (50.0)
Female	5 (50.0)	5 (50.0)	5 (50.0)	4 (50.0)	4 (50.0)
Ethnicity, *n*(%）					
Han nationality	9 (90.0)	9 (90.0)	10 (100.0)	6 (75.0)	6 (75.0)
Other	1 (10.0)	1 (10.0)	0 (0.0)	2 (25.0)	2 (25.0)
Height (cm）					
Mean (SD)	167.60 (7.007)	162.60 (12.635)	162.60 (6.782)	165.06 (9.466)	162.56 (7.164)
Median	168.75	159.25	160.50	165.75	160.50
Weight (kg）					
Mean (SD)	62.20 (6.714)	58.77 (11.392)	60.20 (6.235)	61.26 (6.361)	58.98 (7.860)
Median	63.95	57.20	57.80	61.30	57.95
BMI (kg/m^2^）					
Mean (SD)	22.16 (2.138)	22.05 (1.657)	22.71 (1.119)	22.65 (3.301)	22.30 (2.422)
Median	21.65	22.10	22.55	21.65	21.50

^*a*^ SD, standard deviation; BMI, body mass index; kg, kilogram; *N*, number of subjects in the full analysis set by study arm; *n*, number of subjects in the specified category; %, *n*/N*100.

### Pharmacokinetic result

**Fig 2:**
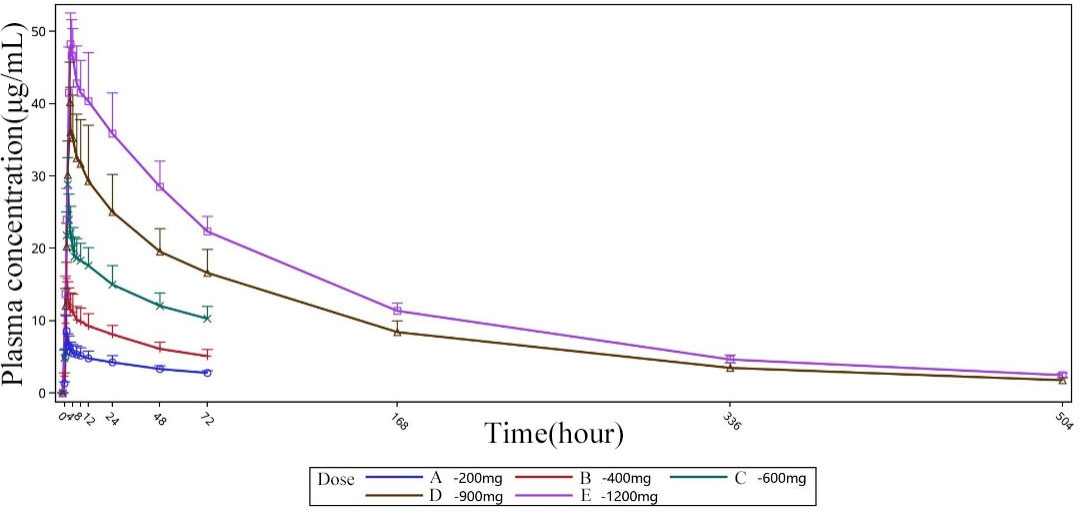
Mean plasma concentration-time profile of HRS9432(A). Single and multiple administrations, groups A to E. Mean, geometric mean; SD, standard deviation.

**Fig 3:**
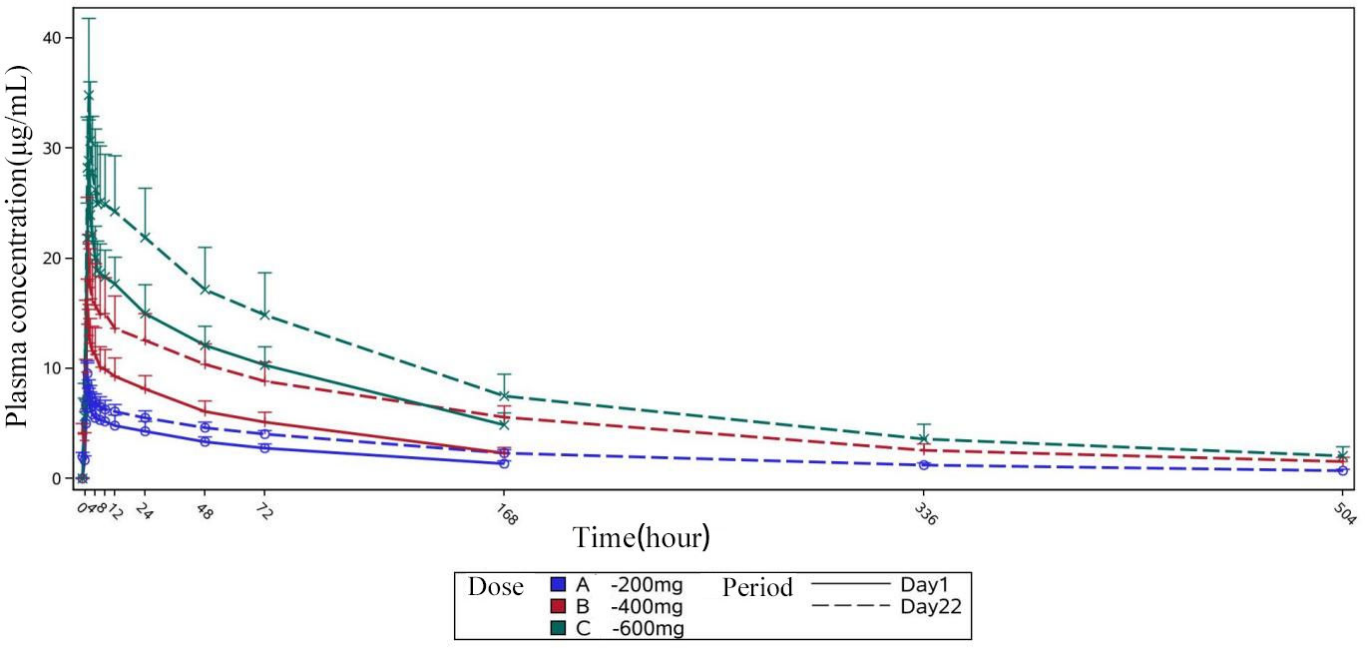
Mean plasma blood concentration-time profile of HRS9432(A). Single administrations, groups A to C. Mean, geometric mean; SD, standard deviation.

Following intravenous infusion of HRS9432(A) in all dose groups, the plasma concentration of HRS9432 peaked immediately at the end of the infusion. The median *T*_max_ for single/multiple dosing was about 1 h in the 200–400 mg group, and 1.5 h in the 600 mg group. For single dosing, the median *T*_max_ was approximately 2.4 h in the 900 mg group and 3.2 h in the 1,200 mg group. Fig. 2 and 3 display the serum concentration-time profile of HRS9432(A).

Both the *C*_max_ and AUC of HRS9432 increased proportionally with the dose in the range of 200–1,200 mg for single administration and 200–600 mg for multiple administrations. After single administration of 900 and 1,200 mg, the *V*_Z_ values were 41.3 and 39.5 L, CL values were 0.194 and 0.203 L/h, and *t*_1/2_ values were 142 and 141 h, respectively. These findings support the feasibility of once-weekly dosing in clinical practice. The PK parameters for single and multiple doses are summarized in Tables 2 and 3, respectively.

**TABLE 2:** HRS9432(A) PK parameters after 200–1,200 mg single administration-Mean (%CV)

Parameter (unit)	A: 200 mg (*N* = 10)	B: 400 mg (*N* = 10)	C: 600 mg (*N* = 10)	D: 900 mg (*N* = 8）	E: 1,200 mg (*N* = 8）
*C*_max_ (μg/mL)	8.63 (23.9)	15.8 (14.5)	28.9 (12.9)	40.2 (14)	49.6 (8.3)
*T*_max_ (h)^*a*^	1.05 (1.00, 1.08)	1.02 (0.983, 1.03)	1.48 (1.42, 1.52)	2.44 (2.42, 2.45)	3.21 (2.90, 4.00)
AUC_0-*t*_ (μg h/mL)	/	/	/	4,186 (17.3)	5,722 (8.8)
AUC_0-*τ*_ (μg h/mL)	473 (16.1)	873 (17.1)	1,709 (16.4)	2,817 (18.1)	3,870 (10.4)
AUC_0-∞_ (μg h/mL)	/	/	/	4,551 (17.8)	6,229 (8.3)
*t*_1/2_ (h)	/	/	/	142 (10.3)	141 (13.4)
*V*_z_ (L)	/	/	/	41.3 (15.4)	39.5 (18.2)
CL (L/h)	/	/	/	0.203 (17.6)	0.194 (8.3)
*λ*_z_ (1 /h)	/	/	/	0.00493 (10.6)	0.00501 (14.7)
MRT_0-*t*_ (h)	64.4 (2.4)	62.7 (2.9)	64.4 (2.4)	139 (5.4)	138 (5.3)
MRT_0-∞_ (h)	/	/	/	184 (8.5)	185 (8.9)
AUC__%Extrap_ (%)	/	/	/	7.92 (17.3)	8.18 (17.8)

^*a*^ *T*_max_ use median (min–max). /, group A–C single administration due to insufficient terminal elimination phase data points; *t*_1/2_, *V*_z_, λ_z_, CL, MRT_0-∞_, AUC_0-∞_, and AUC__%Extrap_ were not counted. %CV, coefficient of variation; Mean, geometric mean; *N*, number of subjects in PK set by study arm.

**TABLE 3:** HRS9432(A) PK parameters after four consecutive 200–600 mg administration—Mean (%CV)^*c*^

Parameter (unit)	A: 200 mg (*N* = 7)	B: 400 mg (*N* = 10)	C: 600 mg (*N* = 10)
*C*_max_ (μg /mL)	9.51 (10.1)	21.3 (19.9)	34.8 (20.1)
*C*_min_ (μg/mL)	2.02 (16.1)	4.02 (24.2)	6.65 (30.1)
*C*_avg_ (μg/mL)	3.99 (9.7)	9.07 (18.9)	14.7 (22.6)
*T*_max_ (h)^*a*^	1.02 (0.983, 1.05)	1.00 (0.967, 1.03)	1.48 (1.43, 1.52)
AUC_0-*t*_ (μg h/mL)	1,108 (12.2)	2,520 (19.1)	3,811 (26.5)
AUC_0-∞_ (μg h/mL)	1,317 (14.7)^*b*^	2,900 (19.2)	4,333 (30.2)
AUC_0-*τ*_ (μg h/mL)	670.3 (9.7)	1,526 (19)	2,463 (22.7)
*t*_1/2_ (h)	187 (11.4)^*b*^	167 (10.3)	166 (19.7)
R_ac, AUC_	1.41 (10.2)	1.75 (5.4)	1.43 (9.2)
R_ac, C max_	1.11 (14.1)	1.34 (7.7)	1.2 (12.3)
*λ*_z_ (1 /h)	0.00374 (11.3)^*b*^	0.00419 (10.3)	0.00433 (20.4)
*V*_z_ (L)	79.9 (8.0)^*b*^	65.7 (25.2)	59.2 (16.2)
CL (L/h)	0.301 (9.9)	0.271 (18.6)	0.254 (19.8)
MRT_0-∞_ (h)	226 (8.8)^*b*^	222 (9.8)	190 (12.1)
DF (%)	189 (12.1)	191 (9.7)	194 (12.6)
AUC__%Extrap_ (%)	14.9 (13)^*b*^	13.1 (18.6)	11.3(28.8)

^*a*^ *T*_max_ use median (min–max).

^*b*^ *N* = 6（one subject had an AUC_%Extrap of 20.45%, and *t*_1/2_, *V*_z_, *λ*_z_, MRT_0-∞_, AUC_0-∞_, and AUC__%Extrap_ were not included in the descriptive summary after this administration).

^*c*^ %CV, coefficient of variation; Mean, geometric mean; *N*, number of subjects in PK set by study arm.

After four consecutive doses of once-weekly dosing, the plasma concentrations of HRS9432 in the 200 and 400 mg groups had not reached steady-state levels, whereas the 600 mg group had achieved a steady-state concentrations. In the dose range of 200 to 600 mg, the relative accumulation ratios (*R*_ac_) for *C*_max_ and AUC_0-τ_ for the fourth dose compared to the first dose ranged from 1.11 to 1.34 and 1.41 to 1.75, respectively. The results of the steady-state analysis are provided inTable 4.

**TABLE 4:** Multiple-dose steady-state analysis^*a*^

Dosage group	Blood collection point	*C*_min_ mean (μg/mL）	*F* value	*P* value
A: 200 mg	D15 (*n* = 9)	1.62	8.43	0.0022
	D22 (*n* = 7)	2.02		
	D29 (*n* = 7)	2.29		
B: 400 mg	D15 (*n* = 10)	3.48	14.17	0.0001
	D22 (*n* = 10)	4.02		
	D29 (*n* = 10)	5.58		
C: 600 mg	D15 (*n* = 10)	5.61	2.47	0.1031
	D22 (*n* = 10)	6.65		
	D29 (*n* = 10)	7.48		

^*a*^ ANOVA based on trough concentrations, with blood collection point as a fixed effect.

### Linear pharmacokinetic analysis

Based on PKPS, the CI method was used to analyze the linear regression of the natural logarithm-transformed exposures, including *C*_max_ and AUC_0-τ_ for single administration and *C*_max_, AUC_0-t_, and AUC_0-∞_ for multiple administrations, with the natural logarithm-transformed administered doses. Single administrations of HRS9432 ranging from 200 to 1,200 mg exhibited a linear regression equation between *C*_max_ and AUC_0-∞_. The slope(β) and 90% CI of the dose-dependent linear regression equations for HRS9432 were 1.02 (0.96–1.09) and 1.21 (1.14–1.28), respectively, indicating that *C*_max_ increased in equal proportions to the dose, while the increase in AUC_0-τ_ was slightly higher than the dose. After multiple administrations of 200–600 mg, HRS9432 exhibited dose-dependent linear regression equations for *C*_max_, AUC_0-t_, and AUC_0-∞_ with slopes (β) and 90% CI of 1.17 (1.03–1.30), 1.11 (0.96–1.26), and 1.06 (0.90–1.22), respectively. The proportional increase in *C*_max_ was slightly higher than the dose, while the proportional increase in AUC_0-t_ and AUC_0-∞_ was equal to that of *C*_max_. This indicates that HRS9432 basically conformed to the linear pharmacokinetic characteristics within the dose range of 200–1,200 mg for single administration and 200–600 mg for multiple administrations.

### Gender difference analysis

Comparison of exposure levels between male and female subjects following single (D1) and multiple (D22) doses revealed that males exhibited lower exposure than females, with geometric mean ratios (male/female) for *C*_max_ and AUC ranging from 0.82 to 0.87. After adjusting for body weight, the gender difference diminished, with geometric mean ratios for *C*_max_ and AUC falling within the range of 0.88 to 0.95. Furthermore, there were no significant gender differences in D22 *C*_max_, AUC_0-t_, and AUC_0-∞_. These findings suggest that gender differences are likely weight-related. Given the limited sample size in this study, the specific gender differences and their clinical significance will be continually monitored in subsequent clinical research.

### Safety

In the multiple-dose group, a total of 244 treatment-emergent adverse events (TEAEs) occurred in 36 subjects during the treatment period, resulting in a TEAE incidence of 100% (36/36). Specifically, the incidence of TEAEs was 75, 48, 78, and 43 occurrences for the 200 mg, 400 mg, 600 mg, and placebo groups, respectively. In the single-dose group, 46 TEAEs occurred in 14 subjects, with a TEAE incidence of 70.0% (14/20). Among these, the incidence of TEAEs was 13, 26, and 7 occurrences for the 900 mg, 1,200 mg, and placebo groups, respectively.

Among the TEAEs, 15 moderate events occurred in 12 subjects, while the remaining 275 events were mild. Of the moderate events, 9 cases of “COVID-19” in 9 subjects were determined to be “unlikely related” to the study drug, while the other 6 cases in 3 subjects were considered “possibly related.” These included incidents of sensory reduction, elevated aspartate aminotransferase, anemia, elevated alanine aminotransferase, upper respiratory tract infection, and muscle spasms, each occurring once. Except for 11 instances of “COVID-19” in 11 subjects and 4 instances of “elevated vitamin C” in 21 subjects, deemed “unlikely related” to the study drug, the remaining 275 TEAEs were considered “possibly related” or treatment-related adverse events (TRAEs). All TRAE outcomes were resolved/recovered (242 cases) or unknown (33 cases), with no infusion site-related adverse events and no serious adverse events. The incidence and severity of all TEAEs showed no apparent dose dependency. TEAEs with an incidence exceeding 10% and higher than that in the placebo group are shown in Table 5.

**TABLE 5:** The incidence of TEAEs exceeded 10% and was higher than that in the placebo group (Safety Set)^*a*^

	Multiple-dose group				Single-dose group		
System organ class (SOC)	A: 200 mg (*N* = 10)	B: 400 mg (*N* = 10)	C: 600 mg (*N* = 10)	Placebo (*N* = 6)	D: 900 mg (*N* = 8)	E: 1,200 mg (*N* = 8)	Placebo (*N* = 4)
Preferred term (PT)	*n* (%)	*n* (%)	*n* (%)	*n* (%)	*n* (%)	*n* (%)	*n* (%)
No. of subjects with TEAE	10 (100.0)	10 (100.0)	10 (100.0)	6 (100.0)	5 (62.5)	6 (75.0)	3 (75.0)
Laboratory abnormalities							
Abnormal T-wave in ECG	0 (0.0)	0 (0.0)	0 (0.0)	0 (0.0)	1 (12.5)	1 (12.5)	0 (0.0)
Decreased K^+^	2 (20.0)	0 (0.0)	1 (10.0)	0 (0.0)	0 (0.0)	1 (12.5)	0 (0.0)
Detection of urinary Bilirubin	0 (0.0)	0 (0.0)	0 (0.0)	0 (0.0)	1 (12.5)	0 (0.0)	0 (0.0)
Detection of urinary sediment	2 (20.0)	1 (10.0)	4 (40.0)	1 (16.7)	0 (0.0)	2 (25.0)	0 (0.0)
Detection of urinary protein	6 (60.0)	0 (0.0)	1 (10.0)	1 (16.7)	0 (0.0)	0 (0.0)	0 (0.0)
Detection of urinary ketones	0 (0.0)	0 (0.0)	0 (0.0)	0 (0.0)	0 (0.0)	1 (12.5)	0 (0.0)
Decreased blood magnesium	3 (30.0)	0 (0.0)	0 (0.0)	0 (0.0)	0 (0.0)	0 (0.0)	0 (0.0)
Decreased blood glucose	0 (0.0)	0 (0.0)	0 (0.0)	0 (0.0)	1 (12.5)	0 (0.0)	0 (0.0)
Elevated blood TG	2 (20.0)	1 (10.0)	4 (40.0)	2 (33.3)	2 (25.0)	2 (25.0)	0 (0.0)
Elevated blood UA	0 (0.0)	2 (20.0)	2 (20.0)	0 (0.0)	1 (12.5)	3 (37.5)	1 (25.0)
Elevated ALT	2 (20.0)	1 (10.0)	1 (10.0)	0 (0.0)	2 (25.0)	2 (25.0)	0 (0.0)
Elevated AST	0 (0.0)	0 (0.0)	0 (0.0)	0 (0.0)	1 (12.5)	1 (12.5)	0 (0.0)
Elevated serum phosphate	1 (10.0)	0 (0.0)	2 (20.0)	1 (16.7)	0 (0.0)	1 (12.5)	0 (0.0)
Elevated serum Ca	0 (0.0)	2 (20.0)	0 (0.0)	0 (0.0)	1 (12.5)	0 (0.0)	0 (0.0)
Elevated Na^+^	0 (0.0)	0 (0.0)	2 (20.0)	1 (16.7)	0 (0.0)	0 (0.0)	0 (0.0)
Increased bile acids	0 (0.0)	0 (0.0)	3 (30.0)	0 (0.0)	0 (0.0)	0 (0.0)	0 (0.0)
Positive urine RBC	4 (40.0)	2 (20.0)	4 (40.0)	2 (33.3)	0 (0.0)	0 (0.0)	0 (0.0)
Positive urinary occult blood	5 (50.0)	4 (40.0)	6 (60.0)	2 (33.3)	0 (0.0)	0 (0.0)	0 (0.0)
Positive urine WBC	0 (0.0)	0 (0.0)	0 (0.0)	0 (0.0)	0 (0.0)	1 (12.5)	0 (0.0)
Respiratory and cardiac organ diseases							
Dyspnea	2 (20.0)	1 (10.0)	0 (0.0)	0 (0.0)	0 (0.0)	0 (0.0)	0 (0.0)
Sinus bradycardia	0 (0.0)	0 (0.0)	0 (0.0)	0 (0.0)	0 (0.0)	1 (12.5)	0 (0.0)
Infectious and invasive diseases							
Upper respiratory tract infection	0 (0.0)	2 (20.0)	1 (10.0)	0 (0.0)	0 (0.0)	0 (0.0)	0 (0.0)
COVID-19	9 (90.0)	0 (0.0)	0 (0.0)	2 (33.3)	0 (0.0)	0 (0.0)	0 (0.0)
Viral gastroenteritis	0 (0.0)	0 (0.0)	0 (0.0)	0 (0.0)	0 (0.0)	1 (12.5)	0 (0.0)

^*a*^ *N*, number of subjects in safety set by study arm; *n*, number of subjects in specified category; %, *n*/N*100; TEAE，treatment-emergent adverse event；ECG，electrocardiogram；K+，serum potassium；TG，triglyceride；UA，uric acid；ALT，alanine aminotransferase；AST，aspartate aminotransferase；Ca，calcium；Na+，plasma sodium；RBC, red blood cells；WBC, white blood cells.

## DISCUSSION

This study aimed to assess the safety, tolerability, and PK characteristics of single (900 and 1,200 mg) and multiple (200 mg, 400 mg, and 600 mg) intravenous infusions of HRS9432(A) for injection in healthy Chinese adults. The results showed that HRS9432 concentrations peaked immediately at the end of infusion after both single and multiple intravenous administrations. The increases in *C*_max_ and AUC within this dose range were approximately proportional to the dose, suggesting linear PK characteristics. Inter-individual variability ranged from 8.3% to 23.9% for *C*_max_ and 8.8% to 17.3% for AUC_0-*t*_ in all groups, indicating a mild degree of variability in exposure levels. Comparison of the PK results of HRS9432 with those of the FDA-approved medication rezafungin (6) revealed that both the single and multiple dosing regimens in this study demonstrate d comparable *in vivo* exposure levels. However, the *t*_1/2_ values for HRS9432 are equal to or greater than those of rezafungin.

Early preclinical study results showed that the MIC values of HRS9432 for *Candida glabrata* CG19, *Candida albicans* CAL6, and *Candida krusei* CK2 were 0.125 µg/mL, 0.06 µg/mL, and 0.25 µg/mL, respectively. PK/PD studies conducted in the murine candidemia model induced by *Candida glabrata* CG19 revealed that after intraperitoneal administration of five doses (0.25 mg/kg, 1 mg/kg, 4 mg/kg, 16 mg/kg, and 64 mg/kg), the AUC_0-168h_/MIC were 86 h, 410 h, 1,632 h, 6,720 h, and 26,400 h, respectively. A higher ratio of AUC_0-t_/MIC corresponds to a lower CFU value of Candida in the kidneys, suggesting increased antifungal efficacy. This indicates a significant correlation between AUC/MIC values and *in vivo* efficacy. An AUC0-168h/MIC of 2000 serves as the PK/PD target for clinical efficacy. Through toxicological studies, NOAELs were determined following 4 weeks of intravenous injection of HRS9432 in SD rats and rhesus monkeys, which were 0.5 mg/kg and 5 mg/kg, respectively. In the mouse model of candida infection treated with HRS9432 at 0.125 mg/kg, the mortality rates on days 3, 4, 5, 6, and 7 were 10%, 50%, 80%, 90%, and 100%, respectively; in the 0.25 mg/kg HRS9432 group, mortality rates on days 4, 5, 6, and 7 were 40%, 60%, 70%, and 90%, respectively; however, no animal deaths were observed in the 1–4 mg/kg HSR9432 treatment group (mortality rate of 0%). Consequently, the starting dose (MRSD) for human clinical trials was calculated based on the NOAEL values obtained from the toxicological studies and was established at 200 mg. The *in vitro* pharmacodynamic study and PK/PD simulation results predicted an effective dose range of HRS9432 from 200 to 600 mg. Considering the guideline requirements for evaluating the cardiac safety of new drugs, a single administration of 1,200 mg was designated as the maximum climbing dose for this study. Additionally, considering the preclinical toxicology data, specifically the MTD of rhesus monkeys (100 mg/kg), and clinical study data of the similar product Rezafungin (tolerance observed up to 1,400 mg administration) (6), it was concluded that the safety risk associated with the 1,200 mg dose was manageable, rendering it suitable as the maximum dose.

Laboratory parameters that were abnormal and clinically significant after administration with an overall incidence of >10% included elevated ALT and detected urine protein in the multiple-dose group, as well as elevated ALT, elevated blood triglycerides and elevated AST in the single-dose group. Notably, elevated ALT was observed in all dose groups of HRS9432(A), whereas the placebo group did not exhibit such elevation. There was no apparent dose-dependent relationship between the severity for ALT/AST abnormalities and the maximum post-administration abnormal values, as well as the duration of AEs for ALT and AST. In this study, three subjects experienced suspected infusion reactions within 5 min of drug initiation, manifesting symptoms such as palpitations, respiratory distress, altered sensations, and discomfort in the head. These TEAEs self-resolved within a few minutes without intervention, and their severity was mild. Histamine testing indicated no significant elevation in subjects following administration, suggesting that the mentioned adverse reactions were not histamine-mediated allergic responses. Furthermore, these TEAEs were limited to the low-dose groups (200 and 400 mg) and did not exhibit a dose-dependent relationship in either incidence or severity. About elevated transaminases, the similar echinocandin antifungal drugs, such as anidulafungin (7) and caspofungin (8), have reported adverse reactions related to hepatic dysfunction. In our clinical study, severe adverse hepatic reactions attributed to HRS9432 have not been observed. Considering that HRS9432 is not metabolized by CYP450 enzymes and exhibits minimal inhibition or induction effects on most CYP metabolic enzymes, we postulate that the drug may trigger hypersensitivity or immune reactions, leading to systemic inflammatory responses or autoimmune hepatitis, consequently resulting in mild elevation of transaminases. Additionally, another plausible reason is the occurrence of COVID-19 viral infection in 9 subjects in this study who were also treated with ibuprofen, which may contribute to hepatic injury either through concomitant medication usage or the viral infection itself (9). Currently, hepatic dysfunction and hypersensitivity reactions have been identified as significant potential risks of HRS9432, and these conditions will be closely monitored in future clinical trials.

In conclusion, this trial demonstrated that intravenous administration of HRS9432(A) at single doses ranging from 200 to 1,200 mg and multiple doses ranging from 200 to 600 mg (once a week, administered four times) results in HRS9432 exhibiting essentially linear pharmacokinetic characteristics in the body. For healthy Chinese subjects, there is no apparent dose-dependent relationship observed in the incidence or severity of all TEAEs. Overall, the safety profile is favorable, and the subjects tolerate the treatment well, supporting a clinical dosing frequency of once weekly.

## MATERIALS AND METHODS

### Study design

This study was a randomized, double-blind, placebo-controlled, dose-escalation phase I first-in-human clinical trial. The study adhered to the principles of dose escalation and included five dosing cohorts, with equal representation of male and female subjects. Stratified block randomization was employed, with each dosing cohort stratified by gender. Cohorts A, B, and C involved multiple administrations, with subjects randomized at a 5:1 ratio into the experimental and placebo groups. The respective dosages were 200, 400, and 600 mg, with a once-weekly dosing regimen for a total of four administrations. Cohorts D and E comprised single administrations, with subjects randomized at a 4:1 ratio into the experimental and placebo groups. The respective dosages were 900 and 1,200 mg. A total of 56 healthy subjects were planned for enrollment, and progression to the next dose group is permissible only if the safety assessment on the 15th day following the last administration of the preceding dose group has not met the termination criteria. Random number tables were generated using SAS statistical software (version 9.4 or above), and randomization codes were assigned for subject allocation.

During the study, the subjects were admitted to the clinical trial ward one day prior to each dosing. They consumed a standardized evening meal and subsequently observed a fasting period of at least 10 h. On the day of each dosing session, subjects received intravenous drug administration 1 h after consuming a standard breakfast. Four hours post-initiation of dosing, subjects uniformly consumed a standard lunch. Groups A–C were observed and followed up until the 43rd day, while Groups D and E were observed and followed up until the 22nd day.

### Subjects

Healthy males or females aged 18–45 years old, with a weight of ≥50.0 kg for males and ≥45.0 kg for females, and a body mass index (BMI) between 19.0 and 28.0 kg/m^2^, who do not have plans for reproduction and exhibit good communication skills.

The key exclusion criteria included: a history of drug allergies or drug abuse; a history of diseases or surgeries that could potentially impact the safety of the trials and the pharmacokinetic processes of the administered drugs; receipt of inactivated/attenuated/live vaccinations; use of or potential need for other medications; participation in any clinical trials within the past 3 months; excessive blood loss or blood donation exceeding 400 mL; smoking, alcoholism, or excessive consumption of tea and coffee; clinically significant abnormal clinical examination results; testing positive for human immunodeficiency virus (HIV) antibodies, hepatitis B surface antigen (HBsAg), hepatitis C antibodies, or syphilis antibodies; pregnant or lactating; difficulty with venous blood collection, needle phobia, or other conditions that prevent the completion of the test.

### Pharmacokinetic analysis

#### Methodological validation

This study established an LC-MS/MS method for determining the concentration of HRS9432 and histamine in human plasma. The method was designed to be free from interference by impurities in the plasma, ensuring accurate sample analysis. The standard curve for HRS9432 ranged linearly from 0.0250 to 50.0 μg/mL, demonstrating excellent linearity. The concentrations at high, medium, low-medium, and low levels were 0.0750 µg/mL, 1.00 µg/mL, 15.0 µg/mL, and 40.0 µg/mL, respectively. The precision, both intra-batch and inter-batch, was consistently remained below 4.6%, and for the lower limit of quantification (LLOQ) concentration, the precision values were below 6.8%. The mean matrix factors, normalized by the internal standard, ranged from 89.0% to 95.1%, with a coefficient of variation ≤1.6%. The extraction recovery for HRS9432 was ranged between 98.6% and 100.6%, while the internal standard rezafungin exhibited an extraction recovery of 99.8%.

#### Sample collection

Due to varying durations of administration (60 min for Groups A/B, 150 min for Group C, and 180 min for Group E), the blood sampling time points differed among the groups. Venous blood samples of 4 mL each were collected at specific time intervals for each group:

Groups A–C: First administration: Day 1 at 0 h (within 1 h before the start of administration), 0.5 h after the start (only for Groups A/B), 1 h (immediately after infusion for Groups A/B), 1.5 h (immediately after infusion for Group C), 2 h, 3 h, 4 h, 5 h (only for Group C), 6 h, 8 h, 12 h, 24 h, 48 h, 72 h (total of 13 sampling points). Second and third administrations: D8 and D15, collecting through concentrations within 10 min before the start of administration. Fourth administration: Day 22 at 0 h (within 10 min before the start of administration), 0.5 h after the start (only for Groups A/B), 1 h (immediately after infusion for Groups A/B), 1.5 h (immediately after infusion for Group C), 2 h, 3 h, 4 h, 5 h (only for Group C), 6 h, 8 h, 12 h, 24 h (D23), 48 h (Day 24), 72 h (D25), 168 h (D29), 336 h (D36), and 504 h (D43) (total of 16 sampling points).

Groups D and E: 0 h (within 1 h before the start of administration), 0.5 h after the start, 1 h, 1.5 h (only for Group D), 2 h (only for Group E), 2.5 h (immediately after infusion for Group D, only for Group D), 3 h (immediately after infusion for Group E), 3.5 h (only for Group E), 4 h, 6 h, 8 h, 12 h, 24 h, 48 h, 72 h, 168 h (D8), 336 h (Day 15), and 504 h (D 22) (total of 16 sampling points).

### Safety assessment

Safety assessments included evaluations of adverse events (AEs) and serious adverse events (SAEs), physical examinations, clinical laboratory tests (including blood routine examination, blood biochemistry, routine urine test, and coagulation function), ultrasound examinations of both upper limbs, vital signs (pulse, blood pressure, temperature), and 12-lead electrocardiogram(ECG) examinations.

### Statistical analysis

PK parameter estimation analysis was performed using Phoenix WinNonlin software based on a non-compartmental model. The analyzed PK parameters after the first dose administration (Day 1 dosing for Groups A to E) included AUC_0-t_, AUC_0-τ_, AUC_0-∞_, *T*_max_, *C*_max_, *t*_1/2_, CL _z_, *V*_z_, and more. Additionally, PK parameters following multiple doses (Day 22 dosing for Groups A–C) included *C*_min_, *C*_max, ss_, AUC_0-t, ss_, AUC_0-∞, ss_, *R*_ac_, and fluctuation. Statistical analysis of PK concentration data, generation of concentration-time curves, calculation of PK parameters, and descriptive statistics were performed using Phoenix WinNonlin (8.2). SAS (9.4) was utilized for PK parameter analysis, subjects’ demographic data, and safety analysis.

The linear relationships between the main pharmacokinetic parameters (*C*_max_ and AUC) and dose were analyzed using the Power Model. The differences in blood concentrations at D15, D22, and D29 before dosing (where D29 represents the concentration at 168 h after dosing from D22) were analyzed by ANOVA to assess steady-state attainment. Employing Pharmacokinetic Parameter Set (PKPS), the natural logarithm transformation of key pharmacokinetic parameters was performed. A multifactorial analysis model was used to analyze gender differences in pharmacokinetics, considering each PK parameter in two analyses: (i) incorporating gender and treatment group and (ii) incorporating gender, treatment group, body weight, and the interaction term of body weight*gender. Geometric means and 95% confidence intervals (CI), along with geometric mean ratios and 90% CI for males relative to females, were estimated for different gender-specific PK parameters. Gender differences in *T*_max_ were assessed using the Wilcoxon rank-sum test.
